# Genome‐scale screening in a rat haploid system identifies *Thop1* as a modulator of pluripotency exit

**DOI:** 10.1111/cpr.13209

**Published:** 2022-03-11

**Authors:** Mei Xu, Yiding Zhao, Wenhao Zhang, Mengyang Geng, Qian Liu, Qian Gao, Ling Shuai

**Affiliations:** ^1^ State Key Laboratory of Medicinal Chemical Biology and College of Pharmacy Nankai University Tianjin China; ^2^ Chongqing Key Laboratory of Human Embryo Engineering Chongqing Health Center for Women and Children Chongqing China; ^3^ Tianjin Central Hospital of Gynecology Obstetrics Tianjin Key Laboratory of Human Development and Reproductive Regulation Tianjin China; ^4^ National Clinical Research Center for Obstetrics and Gynecology Peking University Third Hospital Beijing China; ^5^ Frontiers Science Center for Cell Responses Nankai University Tianjin China

## Abstract

**Objectives:**

The rats are crucial animal models for the basic medical researches. Rat embryonic stem cells (ESCs), which are widely studied, can self‐renew and exhibit pluripotency in long‐term culture, but the mechanism underlying how they exit pluripotency remains obscure. To investigate the key modulators on pluripotency exiting in rat ESCs, we perform genome‐wide screening using a unique rat haploid system.

**Materials and Methods:**

Rat haploid ESCs (haESCs) enable advances in the discovery of unknown functional genes owing to their homozygous and pluripotent characteristics. REX1 is a sensitive marker for the naïve pluripotency that is often utilized to monitor pluripotency exit, thus rat haESCs carrying a *Rex1*‐GFP reporter are used for genetic screening. Genome‐wide mutations are introduced into the genomes of rat *Rex1*‐GFP haESCs via *piggyBac* transposon, and differentiation‐retarded mutants are obtained after random differentiation selection. The exact mutations are elucidated by high‐throughput sequencing and bioinformatic analysis. The role of candidate mutation is validated in rat ESCs by knockout and overexpression experiments, and the phosphorylation of ERK1/2 (p‐ERK1/2) is determined by western blotting.

**Results:**

High‐throughput sequencing analysis reveals numerous insertions related to various pathways affecting random differentiation. Thereafter, deletion of *Thop1* (one candidate gene in the screened list) arrests the differentiation of rat ESCs by inhibiting the p‐ERK1/2, whereas overexpression of *Thop1* promotes rat ESCs to exit from pluripotency.

**Conclusions:**

Our findings provide an ideal tool to study functional genomics in rats: a homozygous haploid system carrying a pluripotency reporter that facilitates robust discovery of the mechanisms involved in the self‐renewal or pluripotency of rat ESCs.

## Graphical abstract

Differentiation of pluripotent rat embryonic stem cells (ESCs) in vitro is difficult to achieve for unknown mechanisms. Rat haploid ESCs (haESCs) have been validated as a powerful tool to target unknown functional genes and pathways based on homozygous genetic screening. Xu et al. utilized *Rex1*‐GFP labelled‐rat haESCs to conduct genome‐scale screening of genes modulating pluripotency exiting. Validation experiments showed that *Thop1* (one of the screened out genes) played very important roles in the random differentiation of rat ESCs in vitro via modulating phosphorylation of ERK.

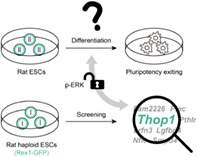

## 1 INTRODUCTION

Rats are ideal animal models for pharmacological and physiological studies due to their suitable size and similarity to humans.^1^ Authentic rat embryonic stem cells (ESCs) can be derived from preimplantation blastocysts with chemically defined 2i/LIF or 3i/LIF medium^2^, ^3^; these cells present a naïve pluripotent state and have the capacity to contribute to the germline. These germline‐competent rat ESCs greatly facilitate the production of transgenic rats because of their stem cell advantages,^4^, ^5^ which are beneficial for developmental and genetic studies in rats. Although rat ESCs show advanced differentiation potential in vivo, their differentiation in vitro is very difficult to achieve because of the severe apoptosis that occurs in this process.^3^, ^6^ This drastically hampers the application of rat ESCs in drug selection research in vitro. To differentiate rat ESCs in vitro, an embryoid body (EB)‐based method and cultivation in conditioned serum medium can be applied to produce differentiated rat cardiomyocytes and neural progenitors.^7^, ^8^ However, these strategies for the differentiation of rat ESCs in vitro are too complicated to be robust, mainly due to the unknown mechanisms underlying self‐renewal or pluripotency. It will be fascinating to investigate the key modulators or pathways involved in the self‐renewal or differentiation of rat ESCs.

Rat haploid ESCs (haESCs), which are recently successfully produced, are novel pluripotent stem cells with only one set of chromosomes. These cells enable the generation of a genome‐scale homozygous mutant library without allelic backups.^9^ Similar to rat haESCs, mouse haESCs can also be used to uncover essential targeting genes of critical biological processes, including pluripotency exit.^10^, ^11^ Although some groups have attempted to supplement the culture medium of rat ESCs to maintain self‐renewal and pluripotency more stably,^12^ even making rat ESCs capable of tetraploid complementation,^13^ the exact mechanisms underlying the effects of these approaches remain unknown. *Rex1* is a sensitive naïve pluripotency marker gene that is often used as a reporter to monitor self‐renewal or pluripotency exit^14^ and is recently found to be suitable for rat ESCs.^15^

In this study, we introduced a *Rex1‐*GFP reporter into rat haESCs and performed high‐throughput genetic screening of random differentiation. Multiple inserted genes and pathways involved in this process were revealed. The findings will be useful for investigating the mechanisms of self‐renewal or pluripotency exit.

## 2 RESULTS

### 2.1 The *Rex1*‐GFP reporter indicates the differentiation of rat HaESCs

**FIGURE 1:**
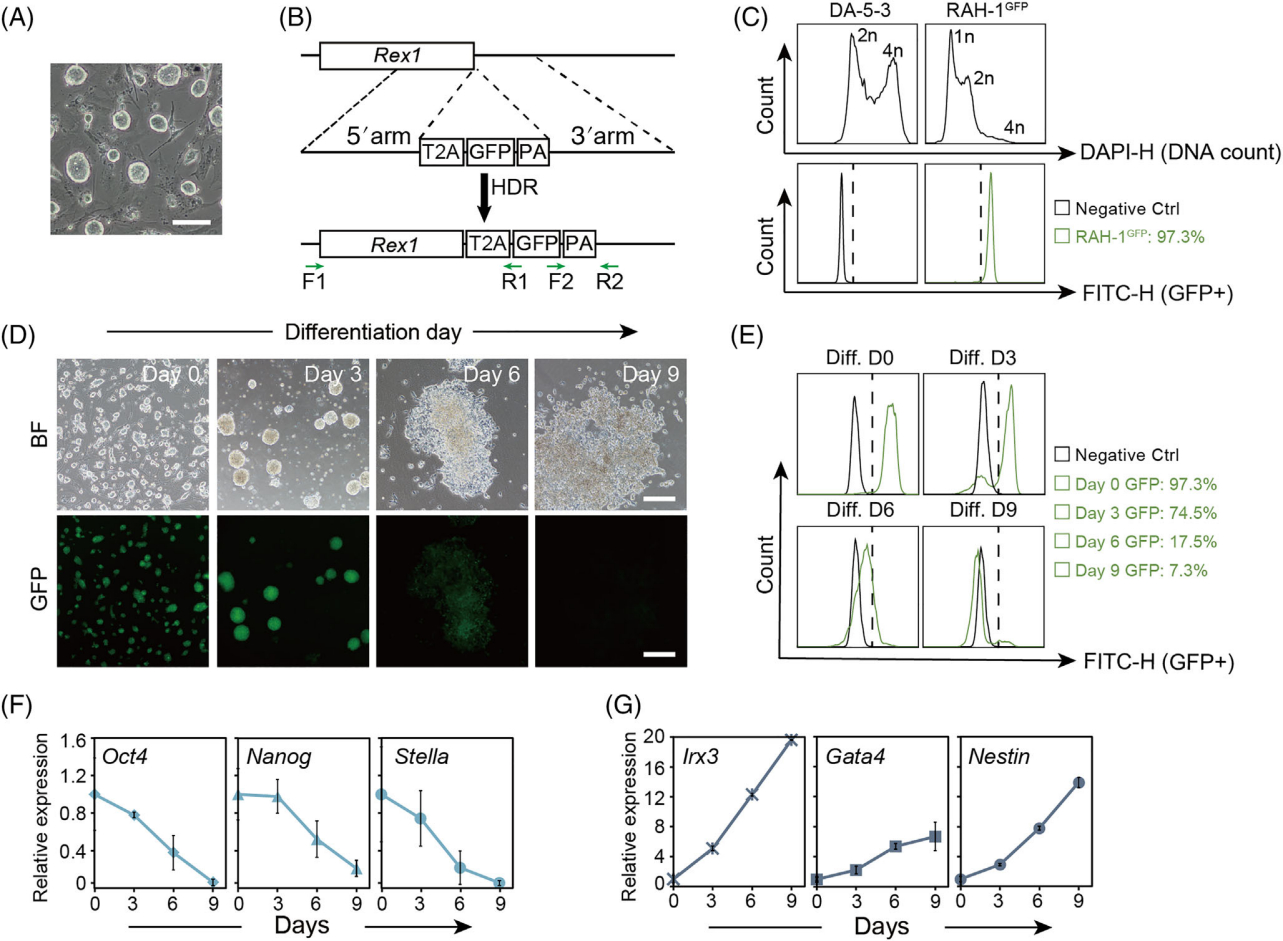
Establishment of rat haESCs carrying the *Rex1‐*GFP reporter. (A) Colonies of rat haESCs in brightfield (BF). Scale bar, 100 μm. (B) Schematic diagram of homologous combination of *Rex1‐GFP* template. (C) DNA content analysis of GFP‐positive cells in RAH‐1^GFP^. The percentage of GFP‐positive cells in RAH‐1^GFP^ was 97.3%, with GFP‐negative DA‐5‐3 (diploid) as a control. (D) BF and FITC‐channel images of cell cultures differentiated from RAH‐1^GFP^ on Day 0, Day 3, Day 6 and Day 9. Scale bar, 100 μm. (E) FACS analysis of GFP‐positive cells in cell cultures differentiated from RAH‐1^GFP^ on Day 0, Day 3, Day 6 and Day 9. The percentages of GFP‐positive cells were 97.7%, 74.5%, 17.5% and 7.3%, respectively. (F) The expression levels of pluripotent genes (*Oct4*, *Nanog* and *Stella*) in cell cultures differentiated from RAH‐1^GFP^ on Day 0, Day 3, Day 6 and Day 9. (G) The expression levels of differentiation genes (*Irx4*, *Gata4* and *Nestin*) in cell cultures differentiated from RAH‐1^GFP^ on Day 0, Day 3, Day 6 and Day 9

**FIGURE 2:**
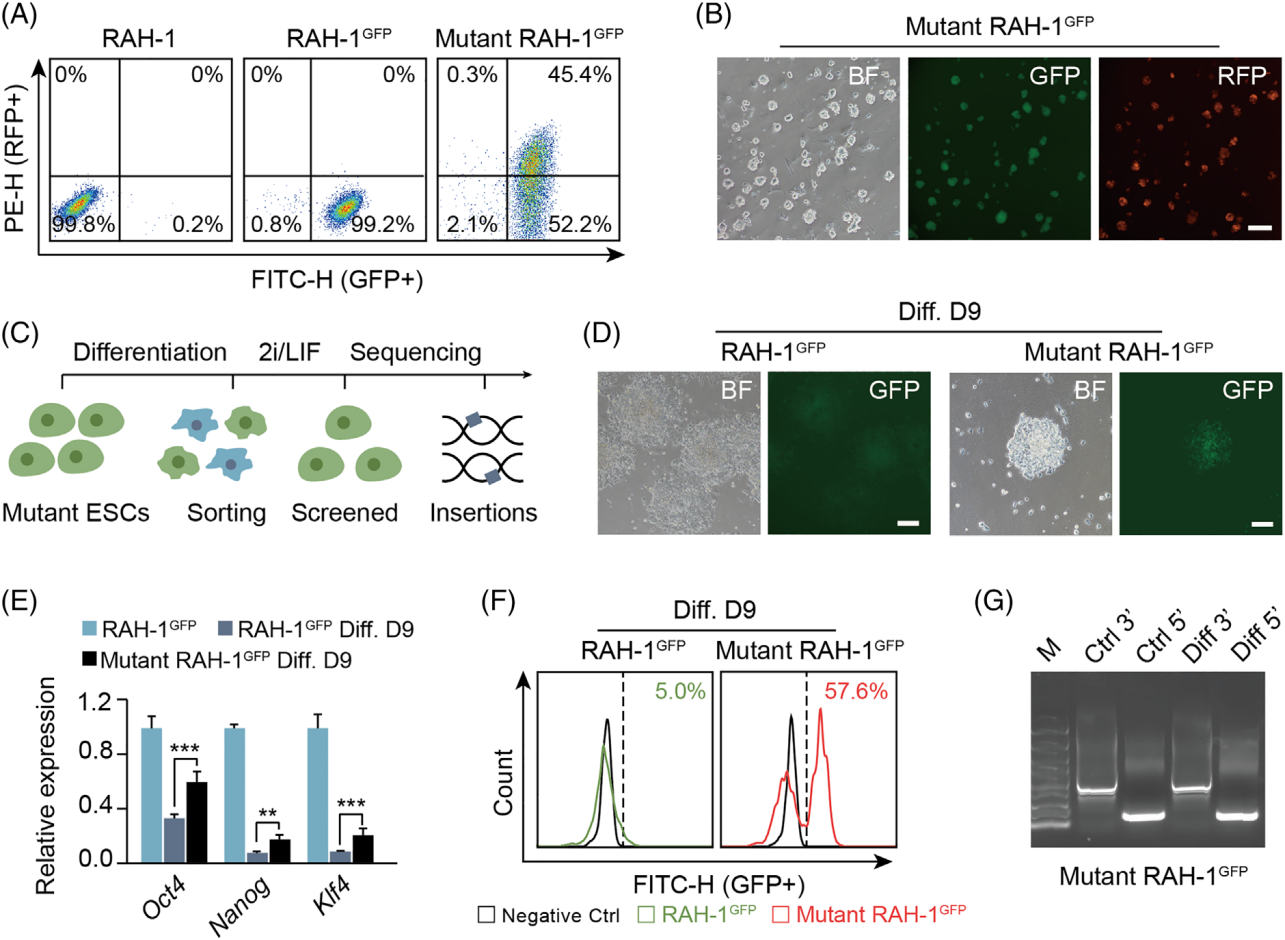
Genetic screening of mutations retarding pluripotency exiting using *Rex1‐*GFP rat haESCs. (A) Enrichment of GFP and RFP double positive (45.4%) cells to obtain mutant RAH‐1^GFP^ by FACS. (B) The images of mutant RAH‐1^GFP^ after sorting in different channels (BF, FITC and TRITC). Scale bar, 100 μm. (C) Schematic overview of the genetic screening of pluripotency exiting with mutant RAH‐1^GFP^. (D) Comparison of differentiated cells on Day 9 between RAH‐1^GFP^ and mutant RAH‐1^GFP^ in BF and FITC‐channel. Scale bar, 100 μm. (E) The expression levels of pluripotent genes (*Oct4*, *Nanog* and *Klf4*) in differentiated cells (from RAH‐1^GFP^ and mutant RAH‐1^GFP^, respectively), with the RAH‐1^GFP^ as a control. *t* test, **p < 0.01, ***p < 0.001. The data were represented as the mean ± SEM. (F) Enrichment of GFP‐positive cells (57.6%) from differentiated cells on Day 9 in mutant RAH‐1^GFP^ group by FACS. FACS of RAH‐1^GFP^ and mutant RAH‐1^GFP^ at differentiation Day 9. (G) Splinkerette PCR products of the screened insertions with controls. Mutant RAH‐1^GFP^ cells before screening were used as controls

We chose a rat haESC (RAH‐1)^9^ cell line with a high percentage of haploids (Figure S1A,B) and standard morphology (Figure 1A) to perform experiments. To construct the *Rex1‐*GFP vector, we designed a template including T2A (a coding sequence of a self‐cleaving linker peptide), a green fluorescent protein (GFP) gene and homologous recombination insertion sites located downstream of *Rex1* (Figure 2B). Next, we electroporated RAH‐1 cells with targeting vectors containing a Cas9‐GFP system and sorted GFP‐positive cells 2 days later for further culturing. After several rounds of enrichment of GFP‐positive cells by fluorescence‐activated cell sorting (FACS), successful homologous recombination of RAH‐1 with GFP downstream of *Rex1* (RAH‐1^GFP^) was achieved. Genotyping PCR further confirmed that RAH‐1^GFP^ carried a *Rex1*‐GFP reporter (Figure S1C). To assess the *Rex1*‐GFP reporter, we further investigated RAH‐1^GFP^ through daily culture and differentiation. Almost every colony of RAH‐1^GFP^ cells presented green fluorescence in long‐term culture when observed in a FITC channel (Figure S1D). The percentage of GFP‐positive cells among RAH‐1^GFP^ cells was as high as 97.3% according to FACS analysis (Figure 1C). Immunostaining for OCT4, NANOG and SSEA‐1 in RAH‐1^GFP^ cells demonstrated that pluripotency was not affected by insertion of the *Rex1*‐GFP reporter (Figure S1E).

Thereafter, we initiated random differentiation of RAH‐1^GFP^ cells according to a previous protocol^7^ with slight modification for 9 days. The green fluorescence in the differentiated cell cultures decreased gradually over time, especially after attachment of EBs (Figure 1D). FACS analysis of the GFP‐positive cells during this process (which decreased from 97.7% to 7.3%) further confirmed this observation (Figure 1E). The quantitative PCR (qPCR) results also suggested that the expression levels of pluripotency genes (*Oct4*, *Nanog* and *Stella*) in differentiated cell cultures decreased over time (Figure 1F), whereas the expression levels of differentiation genes (*Irx3, Gata4* and *Nestin*) increased over time (Figure 1G). Together, these results showed that the *Rex1‐*GFP reporter was a visible tool that could be used to monitor pluripotency and differentiation of rat ESCs efficiently.

### 2.2 Using RAH‐1^GFP^ cells to discover genes restricting pluripotency exit

To achieve genome‐wide mutations, we utilized the PB transposon system to introduce mutations into the RAH‐1^GFP^ cells for subsequent genetic screening. Into a PB vector (PB‐SA‐RFP) carrying a *tdTomato* gene (expressing red fluorescent protein [RFP]) was inserted an SA (splice acceptor) sequence^16^ to improve the efficiency of gene trapping (Figure S2A). To acquire homozygous mutants, we chose RAH‐1^GFP^ cells with a high percentage of haploid cells to perform gene trapping. Approximately 1 × 10^7^ RAH‐1^GFP^ cells were electroporated with the PB‐SA‐RFP and PBase plasmids. Transfected haESCs were further cultured for 5–7 days to stably integrate the PB vectors into the genomes. The cells expressing RFP were mutated by the PB‐SA‐RFP vectors. A total of 45.4% of cells were double‐positive for RFP and GFP in the cell cultures of transfected haESCs, which were sorted by FACS for further culture (Figure 2A). The sorted cells presented double positivity for GFP and RFP by observation (Figure 2B) and were expanded to meet the cell number requirements of next‐generation sequencing (NGS). Thereafter, we conducted random differentiation with these mutant RAH‐1^GFP^ cells as indicated (Figure 2C). Approximately 9 days post differentiation, GFP‐positive cells were still observed among differentiated mutant RAH‐1^GFP^ cells, whereas GFP‐positive cells were only observed among differentiated RAH‐1^GFP^ cells (Figure 2D). qPCR analysis suggested that the expression levels of pluripotency genes (*Oct4*, *Nanog* and *Klf4*) in cells differentiated from mutant RAH‐1^GFP^ cells were significantly higher than those in cells differentiated from RAH‐1^GFP^ cells (Figure 2E). This demonstrated that mutant RAH‐1^GFP^ cells were arrested at differentiation, albeit under random differentiation conditions.

To further investigate the differentiation‐arrested RAH‐1^GFP^ mutants, we harvested GFP‐positive cells (57.6% by FACS) from among cells differentiated from mutant RAH‐1^GFP^ cells and plated them back onto feeder cells with 2i/LIF medium (Figure 2F). Typical rat ESC colonies emerged from the cell cultures of sorted cells nearly 2–3 days later, which we termed the screened library. To analyse the inserted genes preliminarily, we randomly picked 10 subclones from the screened library and performed inverse PCR. Multiple apparent and different insertion fragments were observed after inverse PCR (Figure S2B), which were further sequenced to reveal the inserted genes. Several inserted genes, including *Cacna1b* and *Dact1*, were identified, indicating that mutations involved in random differentiation of rat ESCs could be detected in our system (Figure S2C). Next, we performed splinkerette PCR^17^ with the screened library mixture to prepare samples for NGS. The products of splinkerette PCR showed smeared bands, suggesting that our mutations covered a large proportion of the genome (Figure 2G).

**FIGURE 3:**
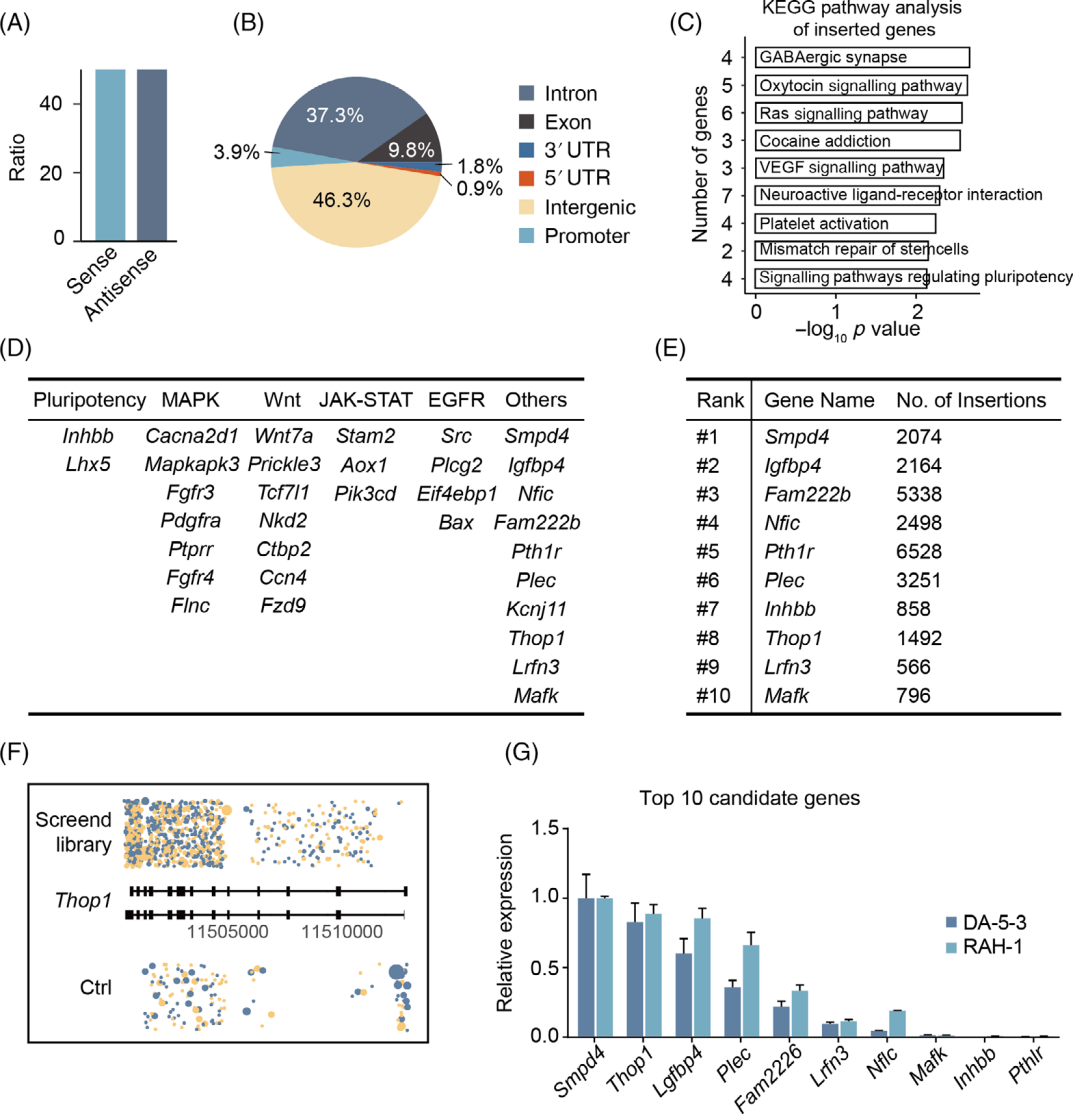
Bioinformatics analysis of insertion sites in the mutant RAH‐1^GFP^. (A) The proportion of the insertional orientations (sense/antisense). (B) The proportion of integration sites across the whole genome. (C) KEGG analysis of the trapped genes mapping to different pathways with frequency. (D) Summary of the top 500 trapped genes mapped to critical pathways related to pluripotency. (E) List of the top 10 trapped genes analysed from the mutant RAH‐1^GFP^. (F) Identified hits located to *Thop1* in screened libraries and control groups. (G) Expression levels of the top 10 trapped genes in DA‐5‐3 and RAH‐1

Our splinkerette PCR products were sent to a local company for NGS, the raw data of which were analysed according to previous reports.^17^, ^18^ According to deep sequencing, approximately 5 million independent insertions located at 18,000 genes were identified. Nearly 50% of the PB‐SA‐RFP vectors were inserted in the sense orientation, and the rest were inserted in the antisense orientation (Figure 3A). A total of 53.7% of the PB‐SA‐RFP vectors integrated into gene bodies (including in the 5′ UTR, 0.9%; promoter, 3.9%; exons, 9.8%; introns, 37.3% and 3′ UTR, 1.8%), and 46.3% integrated into intergenic regions (Figure 3B). An enrichment analysis with KEGG pathway databases revealed that most inserted genes were related to GABAergic synapses, oxytocin signalling pathways, Ras signalling pathways, and so on. (Figure 3C). To comprehensively understand the inserted genes, we analysed them according to previously reported pluripotency‐related pathways (the MAPK, Wnt, JAK–STAT and other pathways). Multiple inserted genes, including *Smpd4*, *Igfbp4*, and so forth, were enriched in these pathways (Figure 3D). To validate the list of inserted genes, we found the top 10 frequently inserted genes (Figures 3E,F and S3A) and analysed their expression levels in rat wild‐type ESCs (WT‐ESCs, DA‐5‐3)^19^ and RAH‐1 cells by qPCR analysis. The qPCR results showed that *Thop1* was highly expressed in both DA‐5‐3 and RAH‐1 cells (Figure 3G), which has not been reported relative to pluripotency to date. We were very interested in addressing whether *Thop1* played essential roles in the pluripotency exit of rat ESCs.

### 2.3 Disruption of *Thop1* retards pluripotency exit in rat ESCs

**FIGURE 4:**
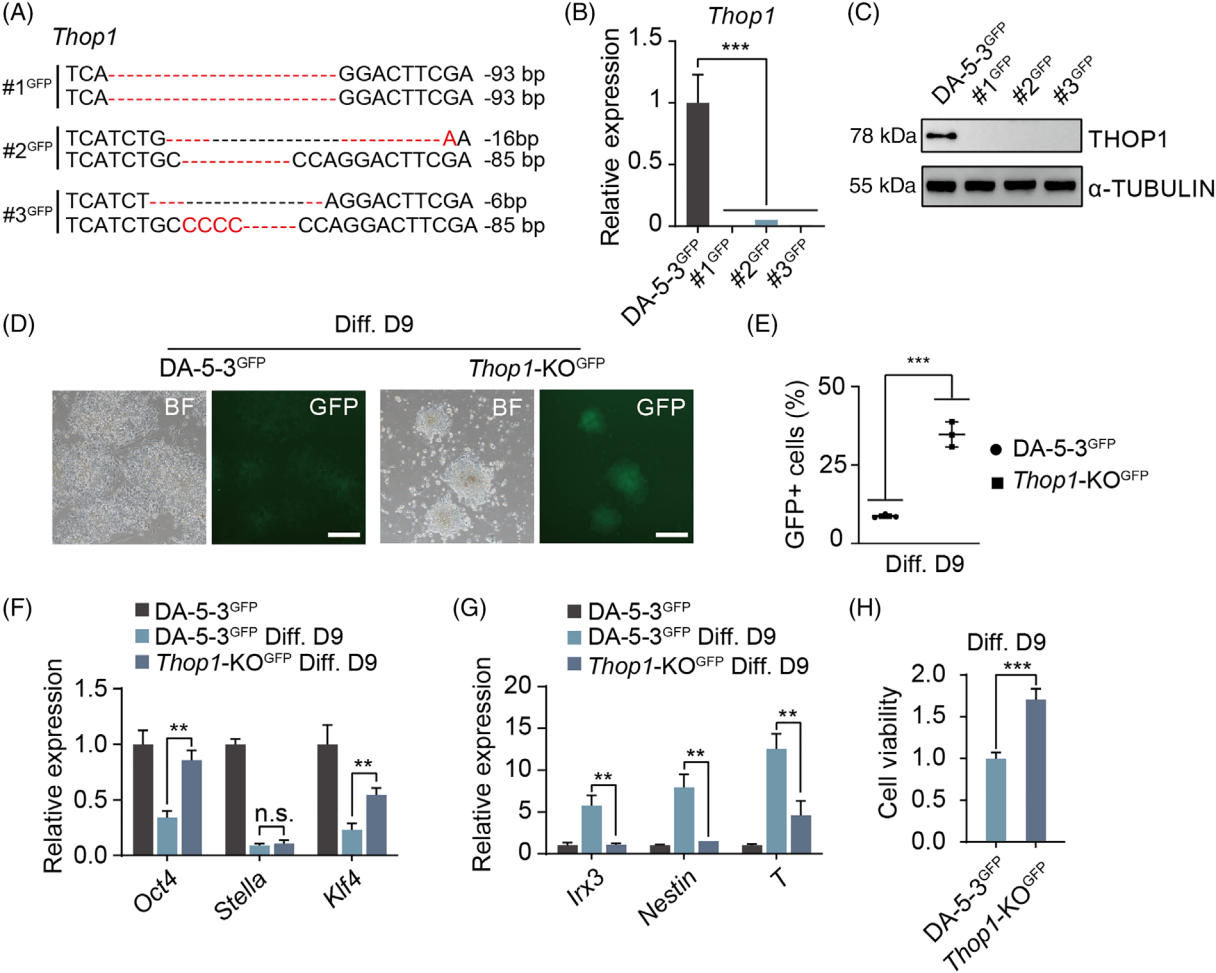
Differentiation of *Thop1*‐KO Rat ESCs. (A) Genotyping analysis of three *Thop1*‐KO subclones (#1^GFP^, #2^GFP^ and #3^GFP^) by DNA sequencing. (B) The expression levels of *Thop1* in #1^GFP^, #2^GFP^ and #3^GFP^ with DA‐5‐3^GFP^ as a control. *t* test, ***p < 0.001. The data were represented as the mean ± SEM. (C) Western blotting analysis of THOP1 in #1^GFP^, #2^GFP^ and #3^GFP^ with DA‐5‐3^GFP^ as a control. α‐TUBULIN was used as a loading control. (D) Comparison of differentiated cells on Day 9 between *Thop1*‐KO^GFP^ and DA‐5‐3^GFP^ in BF and FITC‐channel. Scale bar, 100 μm. (E) FACS analysis of GFP‐positive cells in differentiated cells on day 9. The percentage of GFP‐positive cells in *Thop1*‐KO^GFP^ was 34.7% averagely, while that in DA‐5‐3^GFP^ group was 8.7%, respectively. *t* test, ***p < 0.001. The data were represented as the mean ± SEM. (F) The expression levels of pluripotent genes (*Oct4*, *Stella* and *Klf4*) in differentiated cells on Day 9 (from DA‐5‐3^GFP^ and *Thop1‐*KO^GFP^). DA‐5‐3^GFP^ as control. *t* test, **p < 0.01. The data were represented as the mean ± SEM. (G) The expression levels of differentiation genes (*Irx3*, *Nestin* and *T*) in differentiated cells on Day 9 (from DA‐5‐3^GFP^ and *Thop1‐*KO^GFP^). DA‐5‐3^GFP^ as control. *t* test, **p < 0.01. The data were represented as the mean ± SEM. (H) Cell viability of the differentiated cells from DA‐5‐3^GFP^ and *Thop1‐*KO^GFP^ on Day 9. *t* test, ***p < 0.001. The data were represented as the mean ± SEM

To test whether our candidate inserted genes were related to pluripotency exit of rat ESCs, we chose one of the candidate genes (*Thop1*) to perform proof‐of‐principle validation experiments. *Thop1* is a coding gene encoding thimet oligopeptidase (THOP1), which is mainly expressed in the testes and the brain^20^ and is conserved in many species, including rats.^21^ THOP1 is a zinc metallopeptidase that metabolizes a number of bioactive peptides and degrades peptides released by the proteasome but has not been found to be involved in pluripotency exit. Well grown DA‐5‐3 cells were labelled with the same *Rex1*‐GFP reporter for further investigations (Figure S4A). To delete *Thop1* in DA‐5‐3^GFP^ cells, we transfected DA‐5‐3^GFP^ cells with Cas9‐puro specific sgRNA vectors (Figure S4B). Puromycin‐selected resistant colonies (Figure S4C) were randomly picked, and genotyping was performed. We obtained three *Thop1*‐knockout (KO) subclones with which to perform subsequent experiments (Figure 4A–C). Importantly, *Thop1*‐KO did not affect the *Rex1*‐GFP reporter, as indicated by FACS analysis (Figure S4D). Next, we performed random differentiation with *Thop1‐*KO^GFP^ and DA‐5‐3^GFP^ cells for 9 days as previously described. *Thop1‐*KO^GFP^ cells presented differentiation retardation, and some cells in the cell cultures still expressed GFP on Day 9. In contrast, DA‐5‐3^GFP^ cells did not show this phenotype under the same conditions (Figure 4D). FACS analysis of GFP‐positive cells on Day 9 of differentiation further confirmed this result (Figures 4E and S4E). The qPCR results demonstrated that cells differentiated from *Thop1‐*KO^GFP^ cells had higher expression levels of pluripotency genes (*Oct4* and *Klf4)* and lower expression levels of differentiation genes (*Irx3*, *Nestin* and *T*) than those differentiated from DA‐5‐3^GFP^ cells (Figure 4F,G). In addition, the cell viability of cells differentiated from *Thop1‐*KO^GFP^ cells was also higher than that of cells differentiated from DA‐5‐3^GFP^ cells on Day 9 (Figure 4H). Overall, deletion of *Thop1* could indeed retard the differentiation of rat ESCs.

### 2.4 *Thop1* modulates phosphorylation of ERK1/2 to affect the differentiation of rat ESCs

**FIGURE 5:**
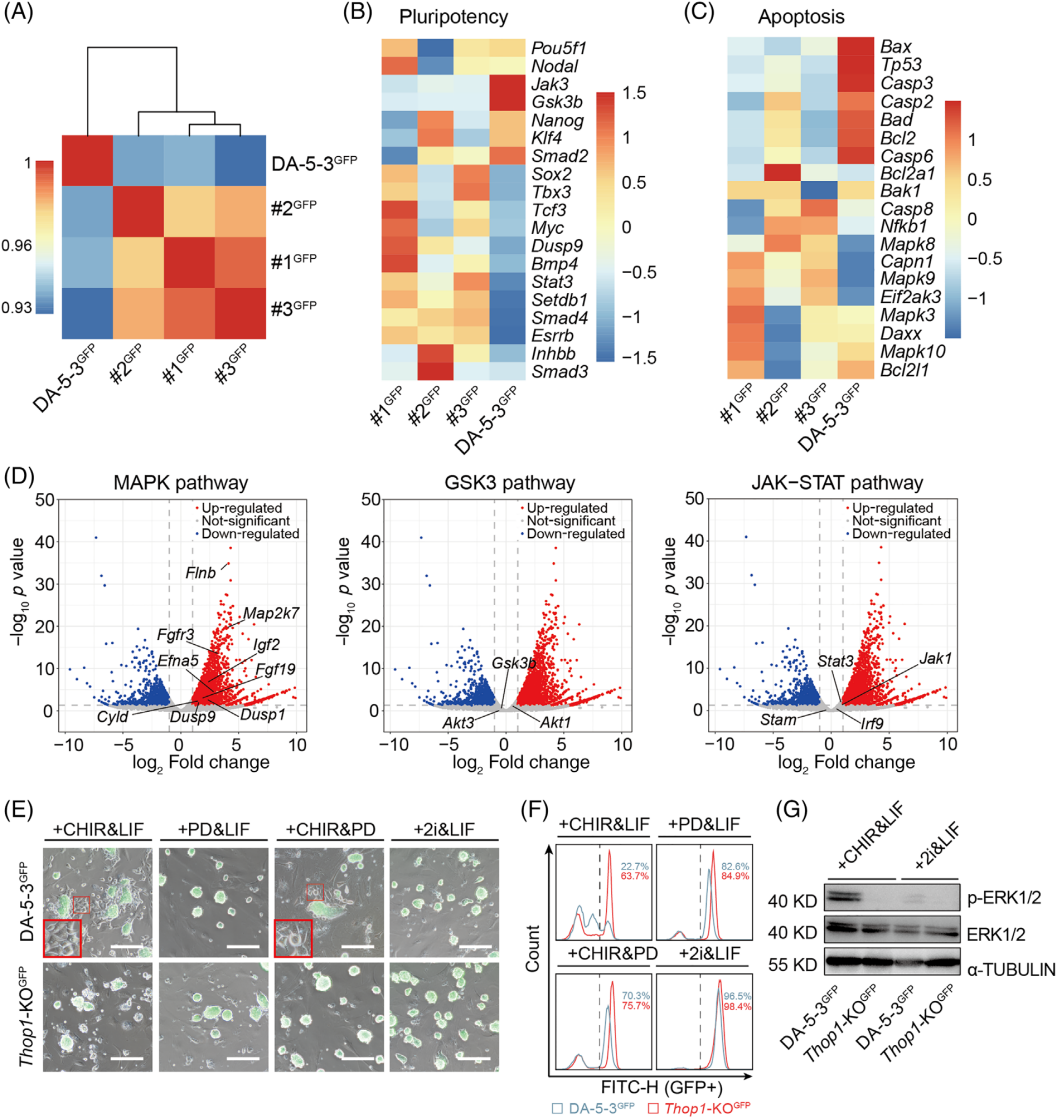
*Thop1*‐KO inhibits phosphorylation of ERK1/2 in the absence of PD0325901. (A) Respective global transcriptome profiles of *Thop1*‐KO^GFP^ (#1^GFP^, #2^GFP^ and #3^GFP^) and DA‐5‐3^GFP^ were hierarchically clustered. (B) The heatmap of representative pluripotent genes among DEGs of *Thop1*‐KO^GFP^ and DA‐5‐3^GFP^. (C) The heatmap of representative apoptosis genes among DEGs of *Thop1*‐KO^GFP^ and DA‐5‐3^GFP^. (D) Volcano plot depicting the differential expression analysis between *Thop1*‐KO^GFP^ and DA‐5‐3^GFP^ and their relationships with the MAPK pathway, the GSK3 pathway and JAK–STAT pathway. Highlight points indicated well‐known genes of the three pathways, considering a fold change of 2.0 and *p* < 0.05. Only the MAPK pathway well‐known genes located to the DEGs. (E) Phase and FITC merged images of DA‐5‐3^GFP^ and *Thop1‐*KO^GFP^ when cultured withdrawing different factors (CHIR&LIF, PD&LIF, CHIR&PD, 2i&LIF) on Day 5. The red arrows indicated the differentiated cells. Scale bar, 100 μm. (F) FACS analysis of GFP‐positive cells in DA‐5‐3^GFP^ and *Thop1‐*KO^GFP^ when cultured withdrawing different factors (CHIR&LIF, PD&LIF, CHIR&PD, 2i&LIF) on Day 5. (G) Western blotting analysis of p‐ERK1/2, ERK1/2 and α‐TUBULIN in DA‐5‐3^GFP^ and *Thop1‐*KO^GFP^ when cultured in 2i/LIF medium or 2i/LIF medium without PD0325901

To determine the changes caused by *Thop1*‐KO, we compared the transcriptomes between *Thop1*‐KO^GFP^ and DA‐5‐3^GFP^ cells by RNA sequencing (RNA‐seq) analysis. The transcriptome levels showed that the three lines of *Thop1*‐KO^GFP^ cells (#1^GFP^, #2^GFP^ and #3^GFP^) were well reproducible and significantly distinct from DA‐5‐3^GFP^ cells (Figure 5A). We further analysed the differentially expressed genes (DEGs) of *Thop1*‐KO^GFP^ and DA‐5‐3^GFP^ cells that were associated with pluripotency and apoptosis. According to the heatmaps, some pluripotency genes (including *Setdb1*, *Esrrb*, etc.) were significantly up‐regulated in *Thop1*‐KO^GFP^ cells (Figure 5B), while some apoptosis genes (including *Bax*, *Tp53*, etc.) were down‐regulated in these cells (Figure 5C). Given that rat *Thop1*‐KO ESCs showed slower differentiation than WT‐ESCs, it was very important to address how *Thop1*‐KO prevented pluripotency exit in rats. We further analysed whether the DEGs of *Thop1*‐KO^GFP^ and DA‐5‐3^GFP^ were involved in the MAPK pathway, the GSK3 pathway and the JAK–STAT pathway (three core pathways related to self‐renewal of rat ESCs). Interestingly, we found that many well‐known genes (*Dusp9*, *Dusp1*, *Cyld*, etc.) from the DEGs located to the MAPK pathway, instead of the GSK3 pathway and JAK–STAT pathway (Figure 5D). We designed more rigorous experiments to determine the relationship of *Thop1*‐KO with 2i (PD0325901 and CHIR99021) and LIF. *Thop1‐*KO^GFP^ and DA‐5‐3^GFP^ cells were cultured in three different media, each lacking one factor of 2i/LIF, for 5 days separately. DA‐5‐3^GFP^ cells exhibited strong differentiation when cultured without PD0325901, whereas *Thop1‐*KO^GFP^ cells did not present obvious differentiation under the same conditions (Figures 5E and S5A). A difference in differentiation was not observed between *Thop1‐*KO^GFP^ and DA‐5‐3^GFP^ cells when they were cultured without CHIR99021 or LIF (Figure 5E). Similarly, FACS revealed that 63.7% of *Thop1*‐KO^GFP^‐positive cells were GFP‐positive, which was much higher than the percentage of DA‐5‐3^GFP^‐positive cells (22.7%) after culture without PD0325901 for 5 days (Figure 5F). However, there was no difference in GFP‐positive cells between the two groups after culture without CHIR99021 or LIF for 5 days. The qPCR analysis suggested that only differentiation without PD0325901 resulted in up‐regulation of all detected pluripotency genes (*Oct4*, *Nanog*, *Klf4*, *Rex1*, *Lin28* and *Sox2*) in *Thop1‐*KO^GFP^ cells compared to DA‐5‐3^GFP^ cells (Figure S5B–D). Therefore, we hypothesized that the effect of *Thop1*‐KO was similar to that of PD0325901. PD0325901 is a MEK inhibitor that suppresses the phosphorylation of ERK1/2 to sustain the self‐renewal and pluripotency of stem cells.^22^, ^23^ We analysed phosphorylated ERK (p‐ERK) in DA‐5‐3^GFP^ and *Thop1‐*KO^GFP^ cells cultured in 2i/LIF medium and 2i/LIF medium without PD0325901. The western blotting results showed that only DA‐5‐3^GFP^ cells cultured without PD0325901 presented obvious p‐ERK levels. In contrast, *Thop1‐*KO^GFP^ cells cultured without PD0325901 and both groups of cells cultured in 2i/LIF medium did not show significant p‐ERK (Figure 5G), suggesting that *Thop1‐*KO was able to inhibit p‐ERK in the same manner as the presence of PD0325901.

**FIGURE 6:**
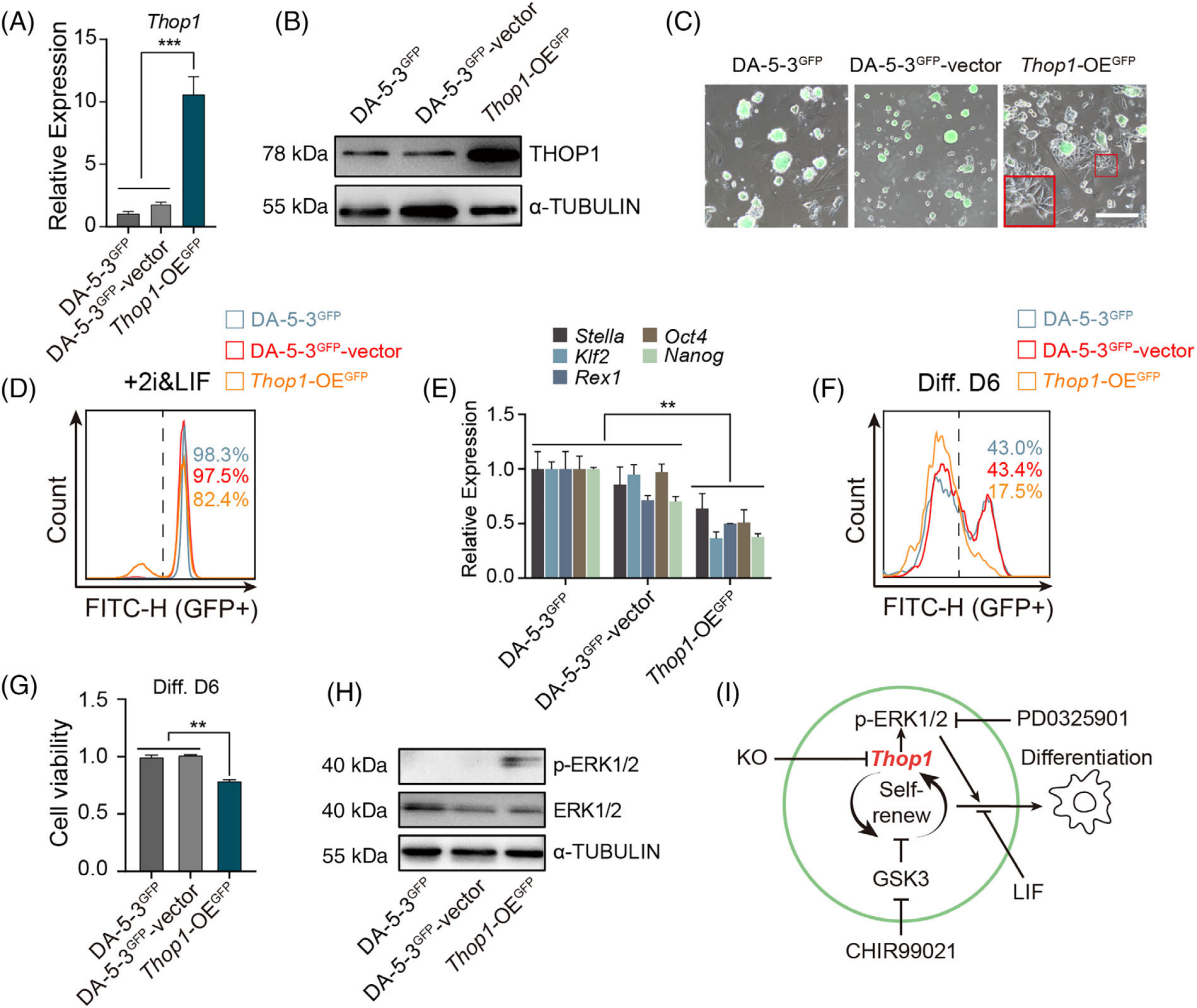
*Thop1*‐OE promotes phosphorylation of ERK1/2. (A) Expression levels of *Thop1* in DA‐5‐3^GFP^, DA‐5‐3^GFP^‐vector and *Thop1‐*OE^GFP^ cells. *t* test, ***p < 0.001. The data were presented as the mean ± SEM. (B) Western blotting analysis of THOP1 and α‐TUBULIN in DA‐5‐3^GFP^, DA‐5‐3^GFP^‐vector and *Thop1*‐OE^GFP^ when cultured in ‘2i/LIF’ medium. (C) Phase and FITC merged images of DA‐5‐3^GFP^, DA‐5‐3^GFP^‐vector and *Thop1*‐OE^GFP^ cells cultured in 2i/LIF medium on Day 3. The red box was an enlarged image of the differentiated cells. Scale bar, 100 μm. (D) FACS analysis of GFP‐positive cells of DA‐5‐3^GFP^, DA‐5‐3^GFP^‐vector and *Thop1‐*OE^GFP^ cells cultured in 2i/LIF medium for 3 days. (E) Expression levels of pluripotent genes (*Klf2*, *Rex1*, *Nanog*, *Oct4* and *Sox2*) in DA‐5‐3^GFP^, DA‐5‐3^GFP^‐vector and *Thop1‐*OE^GFP^ cells cultured 2i/LIF medium for 3 days. *t* test, **p < 0.01. The data were presented as the mean ± SEM. (F) FACS analysis of GFP‐positive cells in the cell cultures of DA‐5‐3^GFP^, DA‐5‐3^GFP^‐vector and *Thop1‐*OE^GFP^ differentiated cells on Day 6. (G) The cell viabilities of DA‐5‐3^GFP^, DA‐5‐3^GFP^‐vector cells and *Thop1‐*OE^GFP^ differentiated cells on Day 6. *t* test, **p < 0.01. The data were represented as the mean ± SEM. (H) The western blotting analysis of p‐ERK1/2, ERK1/2 and α‐TUBULIN in DA‐5‐3^GFP^, DA‐5‐3^GFP^‐vector and *Thop1‐*OE^GFP^ cells cultured in 2i/LIF medium. (I) *Thop1* modulated p‐ERK to affect the differentiation of rat ESCs in vitro

To further analyse the role of *Thop1* in the random differentiation of rat ESCs in vitro, we overexpressed (OE) *Thop1* in DA‐5‐3^GFP^ cells via reconstructed PB‐*Thop1*‐OE vectors, as indicated (Figure S6A). *Thop1*‐OE colonies were enriched by puromycin selection, and DA‐5‐3^GFP^‐vector cells (cells transfected with empty vectors) were used as controls (Figure S6B). Both the qPCR and western blotting results showed that the expression levels of *Thop1* were significantly higher in *Thop1*‐OE^GFP^ cells than in DA‐5‐3^GFP^ and DA‐5‐3^GFP^‐vector cells (Figure 6A,B). During daily culture in 2i/LIF medium, the cell viability of *Thop1*‐OE^GFP^ cells had no difference from those of DA‐5‐3^GFP^ and DA‐5‐3^GFP^‐vector cells (Figure S6C). However, *Thop1*‐OE^GFP^ cells showed obvious differentiation morphology in the cell cultures by observation (Figure 6C). The differentiation of *Thop1*‐OE^GFP^ cells could be further confirmed by FACS analysis of GFP‐positive cells and qPCR (Figure 6D,E). Next, we performed random differentiation with *Thop1*‐OE^GFP^, DA‐5‐3^GFP^ and DA‐5‐3^GFP^‐vector cells for only 6 days. *Thop1*‐OE^GFP^ cells presented more rapid differentiation than DA‐5‐3^GFP^ and DA‐5‐3^GFP^‐vector cells both by observation and FACS analysis on Day 6 (Figure S6D,F). Besides, the cell viability of *Thop1*‐OE^GFP^ differentiated cells was also lower than those of DA‐5‐3^GFP^ and DA‐5‐3^GFP^‐vector differentiated cells on Day 6 (Figure 6G). The western blotting results showed that the expression level of p‐ERK in the *Thop1*‐OE^GFP^ cells increased obviously, compared to those in DA‐5‐3^GFP^ and DA‐5‐3^GFP^‐vector cells when cultured in 2i/LIF medium (Figure 6H), suggesting that *Thop1* OE could promote p‐ERK expression to initiate differentiation in vitro.

## 3 DISCUSSION

Rat haESCs have been widely studied since they were established and can not only give rise to transgenic rats via intracytoplasmic injection^9^ but also be useful in interspecies hybridization research.^24^ Although mouse haESCs have been proven to be powerful tools with which to determine the functions of recessive genes and mutations,^25^ whether rat haESCs are also useful for genetic screening has remained elusive. Here, we performed high‐throughput mutation in rat haESCs using a modified PB transposon system and obtained two independent mutant libraries covering almost the whole genome with millions of insertions (Figure 3A,B). A fragment of SA^26^ constructed into the PB vector was utilized to help us improve the efficiency of mutations (Figure S2A), enabling integration into both exons and introns to generate trapped gene. Combining splinkerette PCR and NGS is a well‐developed strategy to read tremendous amounts of raw data on PB integrations without too much noise.^27^ This strategy was used in our rat haploid system to quickly reveal the outcomes of high‐throughput mutation based on homozygosity. In addition to the transposon mutagenesis strategy, CRISPR sgRNA pools have also been widely utilized to conduct genome‐wide mutation in diploid cells; these pools are also suitable for genetic screening for various purposes, including investigation of pluripotency exit.^28^, ^29^

The key reason for the difficulty of rat ESC differentiation in vitro is that the mechanisms underlying self‐renewal and pluripotency exit are not well known. To address this knowledge gap, we performed genetic screening of pluripotency exit using our rat mutant library. It is difficult to distinguish surviving cells from differentiated rat ESCs, mainly because severe apoptosis occurs during the differentiation process in vitro.^6^, ^7^ This greatly limits the application of rat ESCs in regeneration medicine studies and pharmacological development. Fluorescent reporter genes in ESCs can accurately and sensitively indicate the cell states of living cells in real time and thus are convenient tools for studying the behaviours of ESCs and their derivatives.^30^ Hence, we chose to introduce the *Rex1*‐GFP reporter into rat haESCs and WT‐ESCs to investigate differentiation in vitro visibly and simply. Our data demonstrated that the observation of *Rex1*‐GFP during differentiation in vitro (Figure 1D,E) was coincident with the expression patterns of pluripotency markers and differentiation genes (Figure 1F,G). This ensured the reliability of our genetic screening of pluripotency exit in rats.

Through high‐throughput genetic screening with rat *Rex1*‐GFP haESCs, we revealed multiple inserted genes related to pluripotency that were involved in various pathways (Figure 3D). Particularly, deletion of *Thop1* (one of our found inserted genes) was validated to retard the differentiation of rat ESCs by inhibiting the phosphorylation of ERK1/2. Although we also observed differentiation when cells were cultured without LIF, there was no significant difference between *Thop1*‐KO cells and WT‐ESCs (Figure 5D,E). Whether *Thop1*‐KO has a relationship with the LIF/STAT3 pathway needs more investigation. In addition, overexpression of *Thop1* led to p‐ERK expression in rat ESCs, thus inducing significant differentiation in vitro. Overall, *Thop1* is a pluripotency exit gene for rat ESCs that plays roles in the MAPK pathway (Figure 6F).

There was no direct report that *Thop1* was related to the pluripotency. However, we found that *Dusp1*, *Dusp9* and *Cyld* were up‐regulated in *Thop1*‐KO rat ESCs. *Dusps* family was a critical mediator of BMP signalling to control appropriate ERK activity, being critical for cell fate determination of mouse ESCs.^31^, ^32^ *Cyld* encoded a deubiquitinase cylindromatosis (CYLD), which could inhibit K63 ubiquitination and prevent activation of ERK1 in human cancer cells.^33^, ^34^ All the above results supported that *Thop1* could affect pluripotency exiting through regulating of p‐ERK1/2.

In conclusion, we utilized rat *Rex1*‐GFP haESCs to perform genetic screening and uncovered useful information on pluripotency exit. Mutations including *Thop1* at the genome scale were identified as being related to differentiation. Our findings not only prove that rat haESCs have advantages for use in functional genomics but also shed light on the probable mechanism underlying the self‐renewal and pluripotency of rat ESCs.

## 4 EXPERIMENTAL PROCEDURES

### 4.1 Cell culture and sorting of haploid cells

The rat ESC cell lines RAH‐1 (haploid) and DA‐5‐3 (WT‐ESCs) were kindly donated to us by Dr. Tianda Li and Dr. Qi Zhou (Institute of Zoology, Chinese Academy of Sciences, China). The rat ESCs were cultured on feeder cells in precoated TC plates or TC dishes with previously described 2i/LIF medium^2^ with slight modification. The final concentrations of 2i (PD0325901 [MCE, HY‐10254], CHIR99021 [MCE, HY‐10182]) and human LIF (SinoBiological, 14890‐HNAH) were as follows: PD0325901, 1 μM; CHIR99021, 3 μM and LIF, 1000 U/ml. The medium of the rat ESCs was changed every day, and the cells were passaged every 2–3 days using 0.05% trypsin/EDTA (Thermo, 25300062) with a splicing ratio of 1:4 to 1:6 as needed. For puromycin resistance selection, the concentration of puromycin (Thermo, A1113802) was 1 μg/ml. To enrich haploid cells, RAH‐1 and DA‐5‐3 (diploid control) cells were incubated with 4 μg/ml Hoechst 33342 (Thermo, H3570) for 20 min at 37°C separately and filtered into 5 ml tubes (BD, 352054) with 40 μm cell strainers (BD, 352340). Haploid cells were harvested according to logical gating with a 1n peak on a cell sorter (Beckman, EQ).

### 4.2 Construction of plasmids and electroporation

For the *Rex1‐GFP* template, the 5′ arm and 3′ arm of homologous combination sites in rat *Rex1* and T2A‐GFP‐PA were inserted into the pEASY‐Blunt Simple plasmid (Transgene, CB111‐02). For sgRNA vectors, sgRNAs were designed with the CRISPR design website (http://crispor.tefor.net/) and ligated into pSpCas9n (BB)‐2A‐GFP (PX461) (Addgene, 48140) or pSpCas9(BB)‐2A‐Puro (PX459) (Addgene, 48139). For *piggyBac* (PB)‐SA‐RFP vector construction, an SA fragment was inserted into a PB‐RFP vector,^35^ which was a kind gift from Dr. Wei Li (Institute of Zoology, Chinese Academy of Sciences, China). For overexpression of *Thop1*, the CDS of *Thop1* was inserted into a PB‐CAG‐T2A‐Puro vector (a modified PB dual promoter vector [SBI, cat. no. PB513B]) (Figure S6A). The PBase vector (SBI, PB210PA‐1) was purchased from a local agent. All primers used are listed in Table S1. To deliver vectors into rat ESCs, approximately 2 × 10^6^ cells were electroporated with 5 μg of plasmids using an electroporator (Thermo, NEON) at 1300 V for 10 ms with 3 pulses.

### 4.3 Random differentiation of rat ESCs

Random differentiation of rat ESCs was performed according to a previous protocol^7^ with slight modification. The rat *Rex1‐*GFP ESCs were dissociated to generate single cells and seeded into non‐TC dishes to form EBs. The 2i/LIF culture medium was mixed with conditioned serum medium at a 1:1 ratio. The serum medium contained DMEM (Thermo, 12800017) supplemented with 15% FBS (HyClone, SH30406.05), 100 μM β‐mercaptoethanol (Thermo, 21985023), 1% NEAA (Thermo, 11140050), 2 mM GlutaMAX (Thermo, 35050061) and 100 U/ml penicillin–streptomycin (Thermo, 15140122). Feeder cells were cultured with the serum medium for 1 day, and then the medium was collected as conditioned serum medium. Two days later, the medium of EBs was changed to culture medium (2i/LIF medium mixed with conditioned serum medium) at a 1:3 ratio for another 2‐day culture. On the fifth day, all EBs were seeded in Matrigel (BD, 356230)‐precoated TC plates with serum medium for an additional 5 days of culture. To improve cell viability during the differentiation of rat ESCs, 10 μM Y27632 (MCE, HY‐10071) was added to the medium every day. The proportion of GFP‐positive cells was analysed by FACS on a cell sorter (Beckman, EQ), and fluorescence images were observed on an inverted microscope (Nikon, Ti‐S).

### 4.4 Molecular and cellular analysis and CCK‐8 assay

Immunostaining and karyotype analysis were performed according to a previous report.^36^ The primary antibodies used included anti‐OCT4 (Abcam, ab18976), anti‐NANOG (Abcam, ab80892) and anti‐SSEA‐1 (CST, 4744) antibodies. The secondary antibodies were purchased from Abcam. The nuclei were stained with Hoechst 33342 for 10 min. Immunofluorescence images and chromosome spread images were captured under a confocal microscope (Leica, TCS SP8). The qPCR and western blotting were performed according to our previous work.^37^ All the qPCR primers used are listed in Table S1. The primary antibodies used in western blotting included anti‐THOP1 (Abclonal, A8756), anti‐α‐TUBULIN (Sungene, KM9007T), anti‐ERK1/2 (Abclonal, A4782) and anti‐pERK1/2 (Abclonal, AP0974) antibodies. For the cell viability assay, the differentiated cells were plated into 96‐well plates at a density of 5000 cells per well and cultured with serum medium for 24 h. Then, the cell cultures were incubated with 5 mg/ml buffer from a Cell Counting Kit (CCK‐8) (Yeasen, 40203ES60) for another 4 h. The signals of the culture media were read using a BioTek luminescence reader (Bio‐Rad) at 450 nm.

### 4.5 Inverse PCR and splinkerette PCR

For inverse PCR, the genomic DNA extracted from cells was digested by PSUI (Thermo, FD1554), self‐ligated, and amplified with two rounds of nested PCR.^17^ The PCR products were purified using a PCR product purification kit (Sangon Biotech, B518141) and inserted into the pEASY‐Blunt Simple plasmid (Transgene, CB111‐02) in preparation for Sanger sequencing. The basic steps of splinkerette PCR were similar to those of inverse PCR but with different primers. All primers and splinkerette adaptors used are listed in Table S1. Sanger sequencing was performed by Tsingke, and the splinkerette PCR products for NGS were sent to another local company (Novogene).

### 4.6 Bioinformatic analysis of insertions and RNA‐seq

For the PB‐mutation library, the adapters and PB tags were removed from the read pairs before mapping to the genome using the FASTX toolkit (http://hannonlab.cshl.edu/fastx_toolkit/commandline.html). HaSAPPy^18^ was run to align the trimmed reads to the genome assembly from UCSC (mm10).^38^ We analysed the insertions for each gene with Gencode^39^ with the default parameters.

All the RNA‐seq data were sequenced by a local company (Novogene). The raw data were processed with the FASTX toolkit to remove noise and the adaptors. Genes with an adjusted *p* value <0.05 that were identified by DESeq2 were considered DEGs. The R heatmap function was used to perform hierarchical clustering.

## CONFLICT OF INTEREST

The authors declare no conflict of interest.

## AUTHOR CONTRIBUTIONS

Ling Shuai and Qian Gao conceived, designed and supervised the study. Mei Xu, Yiding Zhao, Wenhao Zhang, Mengyang Geng and Qian Liu performed the experiments. Mei Xu, Yiding Zhao and Ling Shuai wrote this manuscript.

## Supporting information


**Appendix** **S1:** Supporting information.

## Data Availability

The data that support the findings of this study are available from the corresponding author upon reasonable request. Sequence data supporting the findings of this study have been deposited in GEO database, including DNA sequencing experiments with the mutant rat haESC library (GEO: GSE 190480) and RNA sequencing experiments (GEO: GSE 190318).
