# Supplementary material for: Genome‐scale screening in a rat haploid system identifies Thop1 as a modulator of pluripotency exit
Source: Cell Prolif. 2022 Mar 11;55(4):e13209. doi: 10.1111/cpr.13209 (PMC9055895; doi:10.1111/cpr.13209)
Supplement: Supplementary file 1 — Appendix S1: Supporting information. [file CPR-55-e13209-s001.docx]

**Supplementary Materials**

**Title:** Genome-scale Screening in a Rat Haploid System Identifies *Thop1* as a Modulator of Pluripotency Exit

**Running title:** Genetic Screening with Rat Haploid System

**Authors and affiliations:**

Mei Xu^1,6^, Yiding Zhao^1,6^, Wenhao Zhang^1,5,6^, Mengyang Geng^1^, Qian Liu^1^, Qian Gao^1*^ and Ling Shuai^1,2,3,4*^

^1^ State Key Laboratory of Medicinal Chemical Biology and College of Pharmacy, Nankai University; Tianjin 300350, China.

^2^ Tianjin Central Hospital of Gynecology Obstetrics/Tianjin Key Laboratory of Human Development and Reproductive Regulation, Tianjin 300350, China.

^3^ National Clinical Research Center for Obstetrics and Gynecology, Peking University Third Hospital, Beijing 100191, China.

^4^ Frontiers Science Center for Cell Responses, Nankai University; Tianjin 300350, China.

^5^ Chongqing Key Laboratory of Human Embryo Engineering, Chongqing Health Center for Women and Children, Chongqing 400013, China.

^6^ Co-first author.

**^*^ Correspondence:** gaoqian@nankai.edu.cn (Q.G.); lshuai@nankai.edu.cn (L.S.)

**Lead Contact:** lshuai@nankai.edu.cn (L.S.)


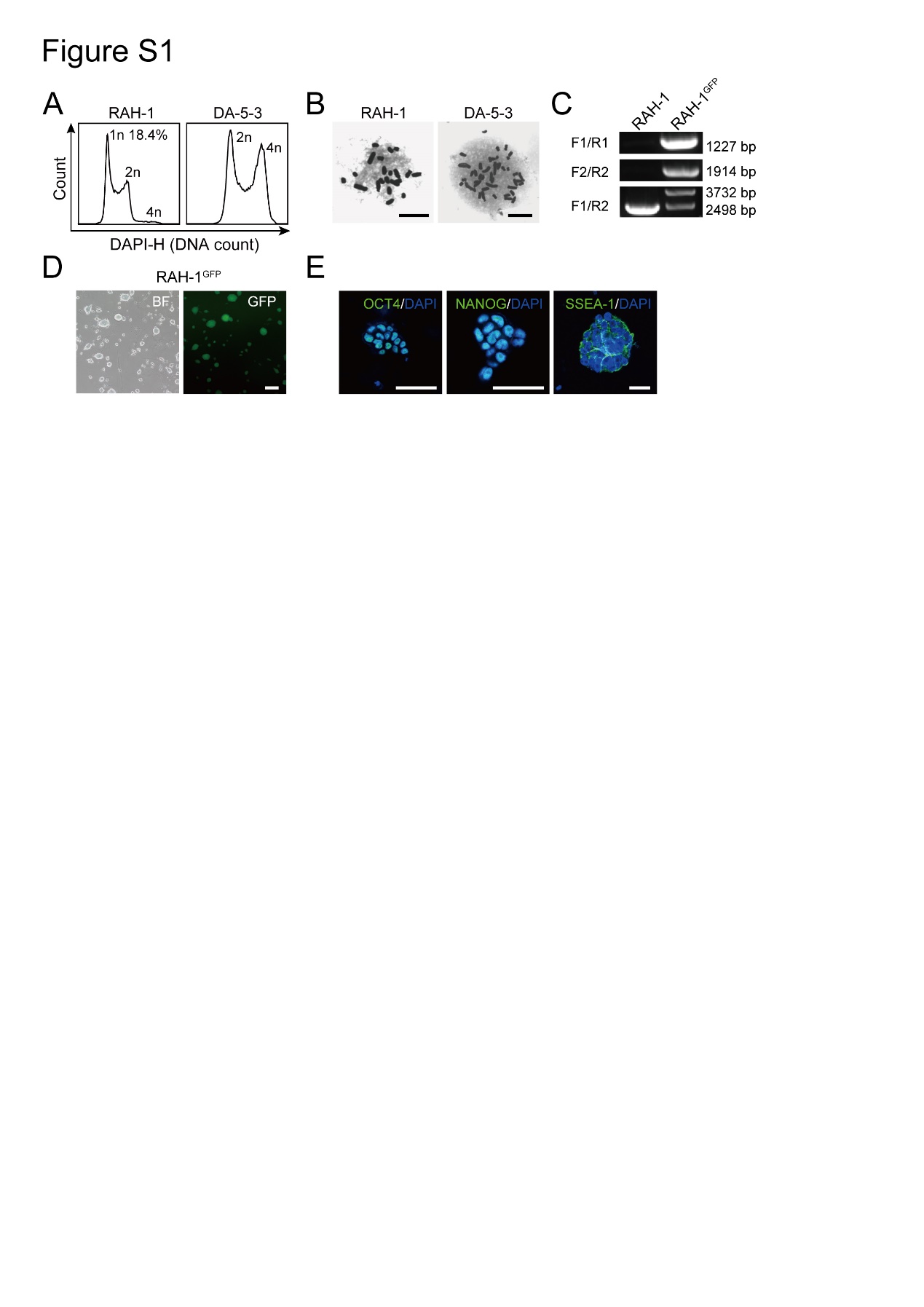


**Figure S1,** **related to Figure 1 - RAH-1 carrying a *Rex1-*GFP reporter.**

(A) DNA content analysis of RAH-1. The percentage of 1n peak (haploid cells at G0/G1 phase) in RAH-1 was 18.4%, with DA-5-3 as a diploid control.

(B) A chromosome spread analysis indicated that RAH-1 had a set of chromosomes (20+X). Scale bar, 10 μm.

(C) Genotyping identified that RAH-1^GFP^ carried a *Rex1-*GFP reporter.

(D) BF and FITC-channel images of RAH-1^GFP^. Scale bar,100 μm.

(E) Immunofluorescence of pluripotent markers (OCT4, NANOG and SSEA-1) in RAH-1^GFP^. DNA was stained with Hoechst 33342. Scale bar, 50 μm.


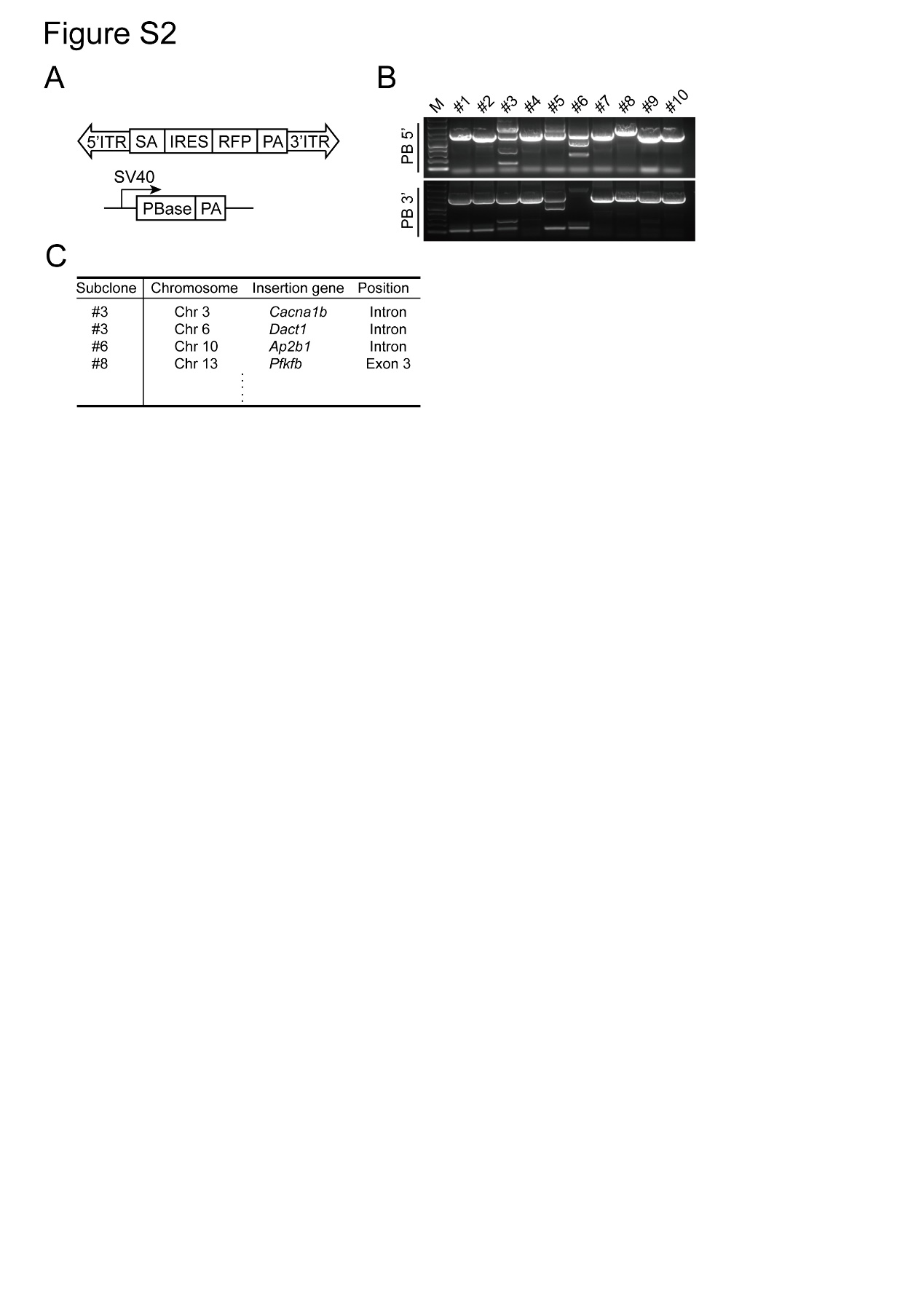


**Figure S2, related to Figure 2 - Analysis of insertion sites.**

(A) Schematic diagram of the plasmids of PB-SA-RFP and PBase.

(B) Inverse PCR validation of the insertion sites among the screened libraries. The different bands presented distinct insertions.

(C) Summary of some PB integration sites identified by inverse PCR.


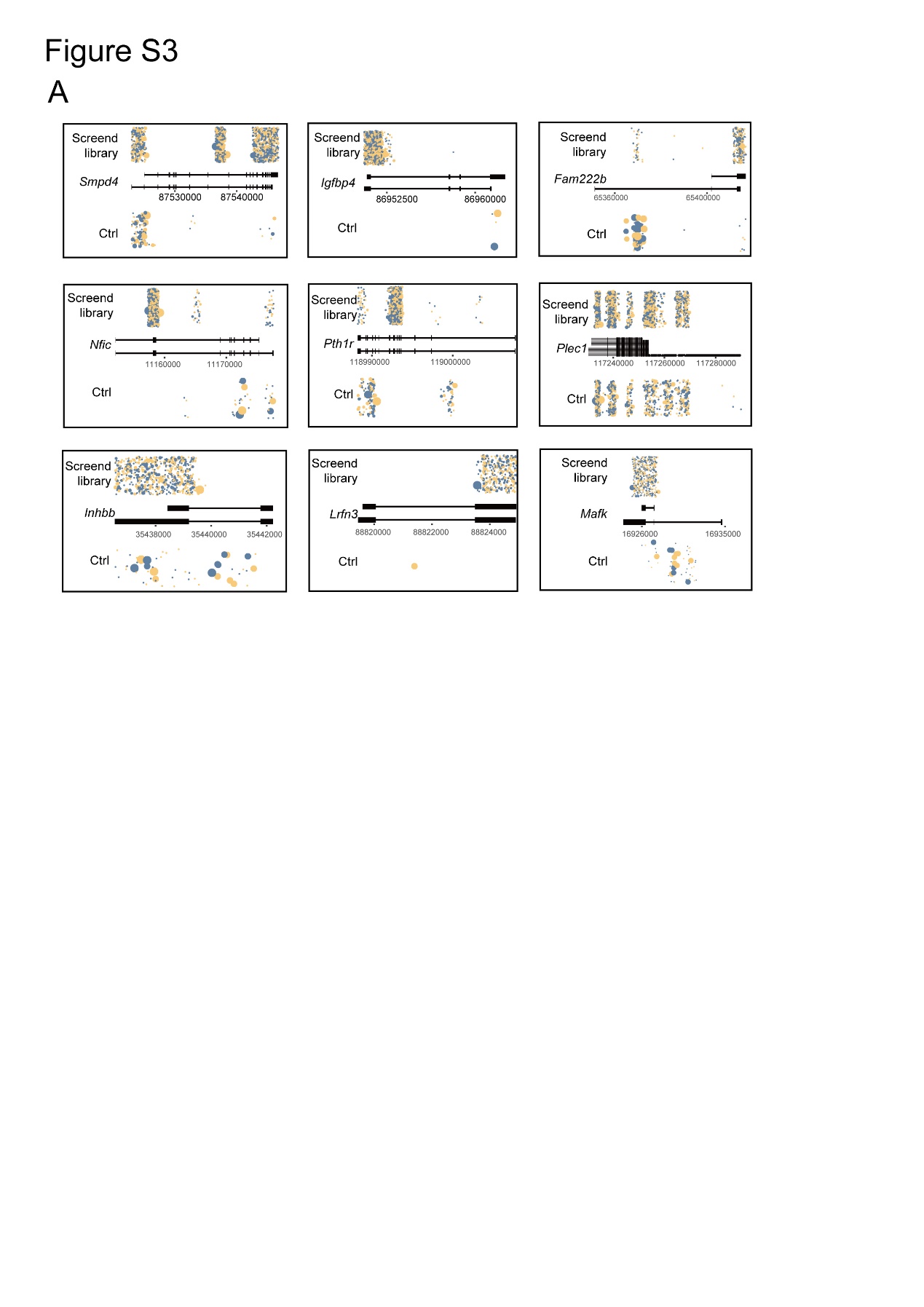


**Figure S3, related to Figure 3 - The enrichments of the top 10 inserted genes in the screened library.**

(A) Strand-specific coverage tracks of the genes including *Smpd4*, *Igfbp4*, *Fam222b*, *Nfic*, *Pth1r*, *Plec1*, *Inhbb*, *Lrfn3* and *Mafk* among the screened library.


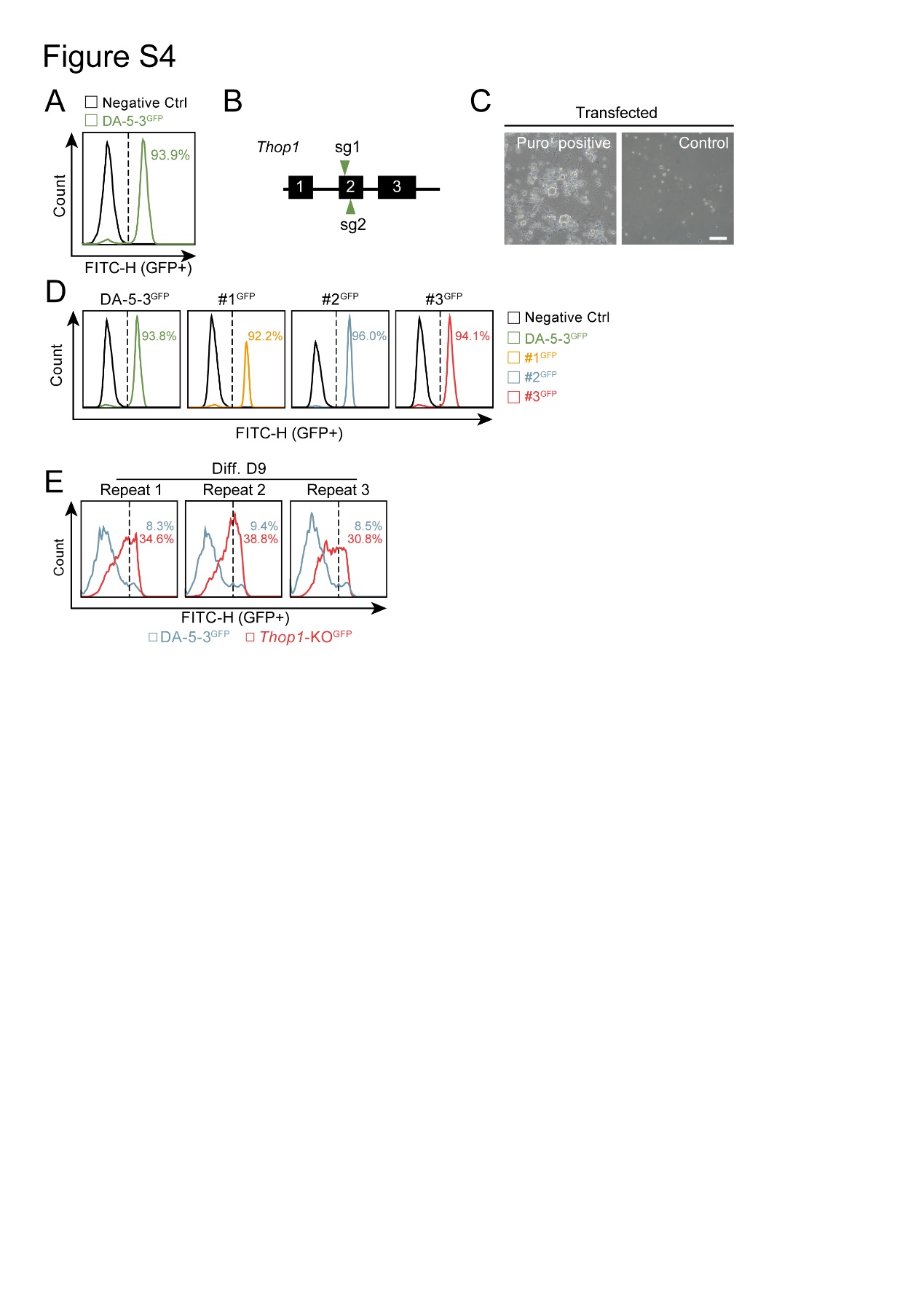


**Figure S4, related to Figure 4 - Rat *Thop1*-KO ESCs showed retard of differentiation in random differentiation.**

(A) The percentage of GFP positive cells in DA-5-3^GFP^, with DA-5-3 as a negative control.

(B) Schematic diagram of CRISPR/Cas9 system to knock out *Thop1*.

(C) DA-5-3^GFP^ transfected with Cas9-sgRNAs were selected for puromycin on day 2. DA-5-3^GFP^ without transfection was used as a control. Scale bar, 100 μm.

(D) The percentage of GFP positive cells in DA-5-3^GFP^ and *Thop1*-KO^GFP^ subclones (#1^GFP^, #2^GFP^ and #3^GFP^), with DA-5-3 as a negative control.

(E) FACS analysis of GFP positive cells in differentiated cells from DA-5-3^GFP^ and *Thop1*-KO^GFP^ on day 9, with DA-5-3 as a negative control.


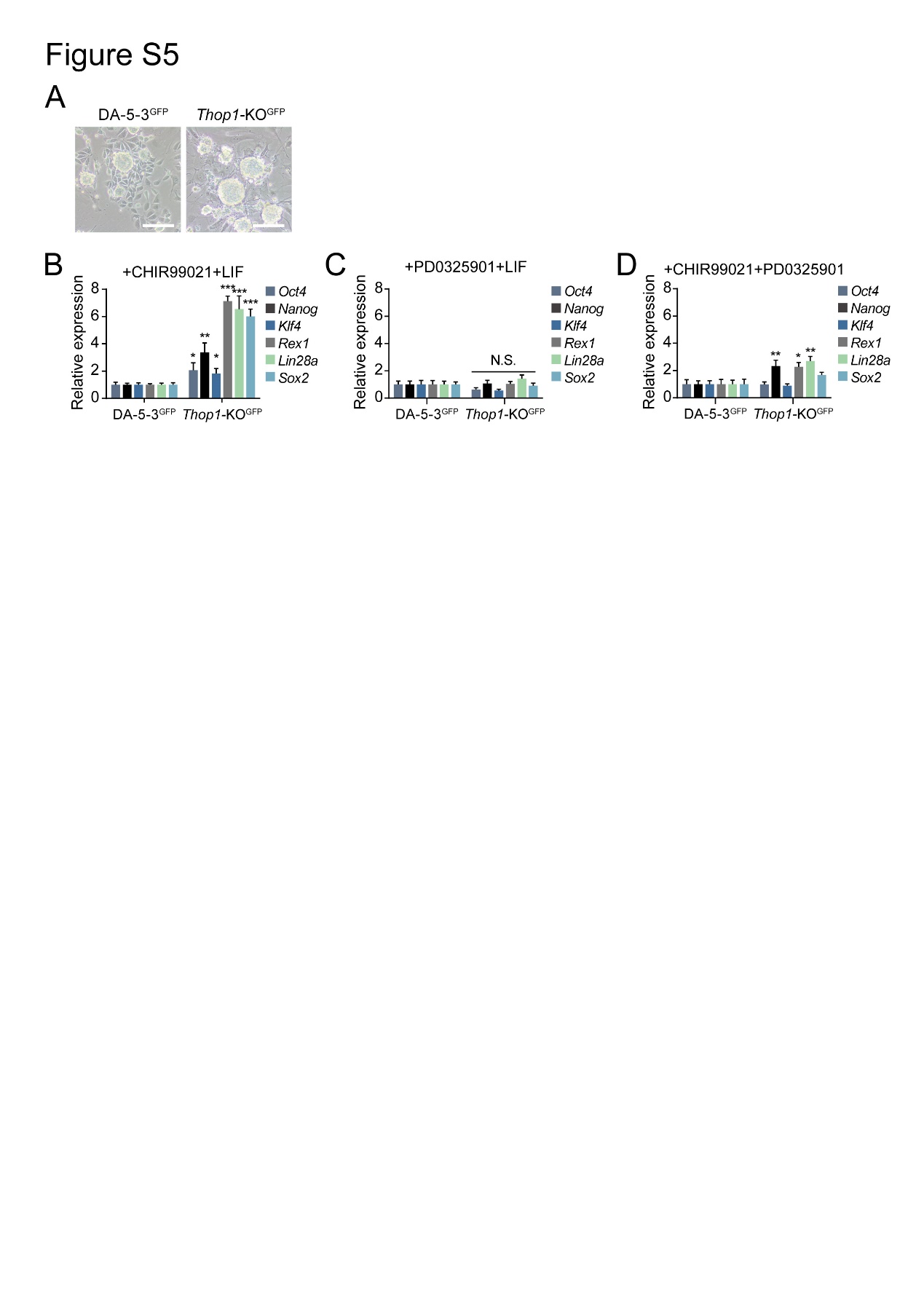


**Figure S5, related to Figure 5 - Rat *Thop1*-KO ESCs showed retard of differentiation when cultured withdrawing PD0325901.**

(A) Phase images of DA-5-3^GFP^ and *Thop1-*KO^GFP^ when cultured withdrawing PD0325901 on day 5. Scale bar, 100 μm.

(B) The expression levels of pluripotent genes (*Oct4*, *Nanog*, *Klf4*, *Rex1*, *Lin28a* and *Sox2*) in DA-5-3^GFP^ and *Thop1-*KO^GFP^ when cultured withdrawing PD0325901 on day 5. t test, *p < 0.05, **p < 0.01, ***p < 0.001. The data was presented as the mean ± SEM.

(C) The expression levels of pluripotent genes (*Oct4*, *Nanog*, *Klf4*, *Rex1*, *Lin28a* and *Sox2*) in DA-5-3^GFP^ and *Thop1-*KO^GFP^ when cultured withdrawing CHIR99021 on day 5. The data was presented as the mean ± SEM.

(D) The expression levels of pluripotent genes (*Oct4*, *Nanog*, *Klf4*, *Rex1*, *Lin28a* and *Sox2*) in DA-5-3^GFP^ and *Thop1-*KO^GFP^ when cultured withdrawing LIF on day 5. t test, *p < 0.05, **p < 0.01. The data was presented as the mean ± SEM.


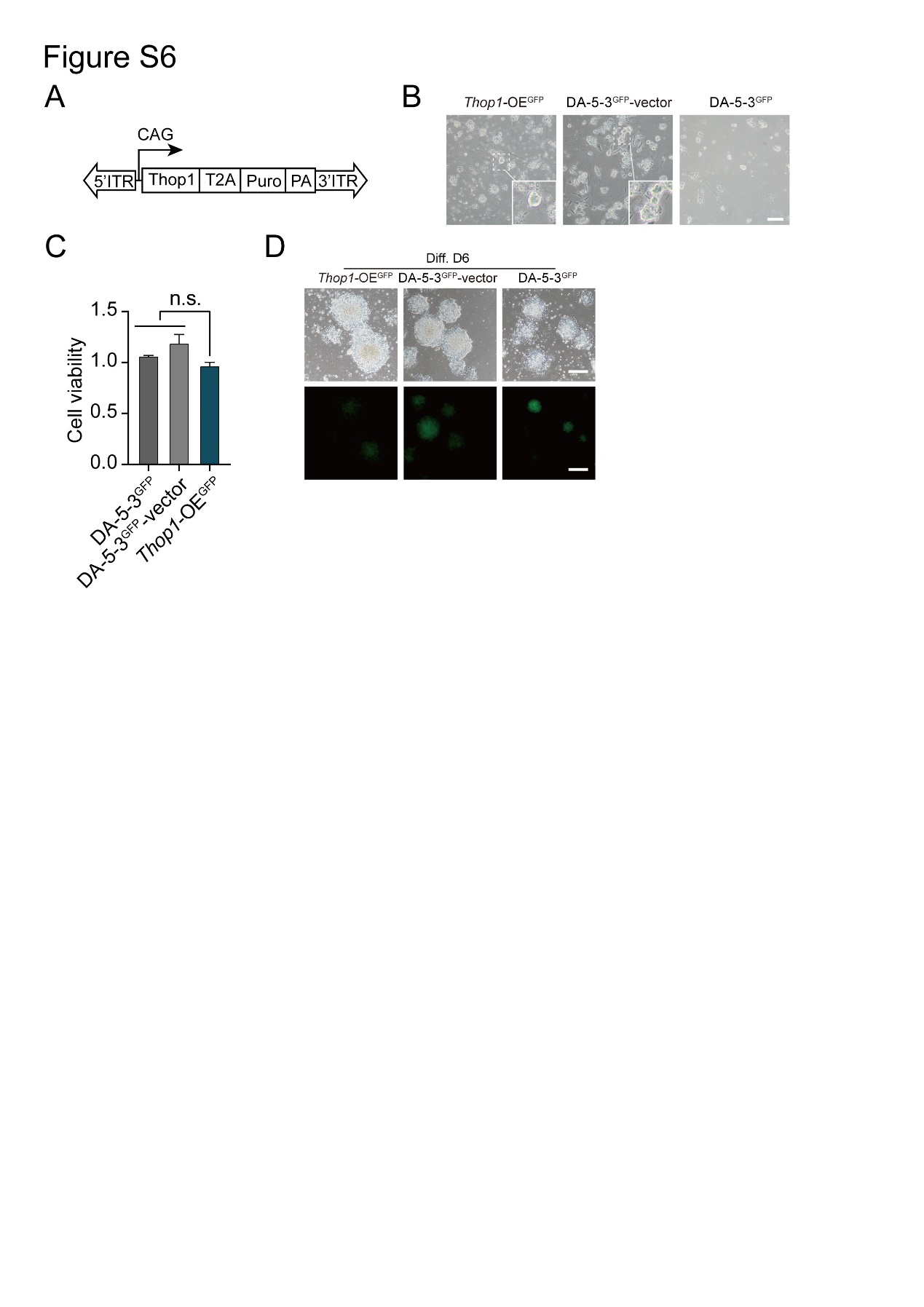


**Figure S6, related to Figure 6 - Overexpression *Thop1* in rat ESCs.**

(A) Schematic diagram of the plasmids of PB-*Thop1*-OE.

(B) DA-5-3^GFP^ transfected with plasmids of PB-*Thop1*-OE and empty vectors were selected for puromycin on day 2. DA-5-3^GFP^ without transfection was used as a control. Scale bar, 100 μm.

(C) Cell viability of DA-5-3^GFP^, DA-5-3^GFP^-vector and *Thop1*-OE^GFP^ cells cultured in 2i/LIF medium on day 3. t test, ***p < 0.001. The data was represented as the mean ± SEM.

(D) Comparison of differentiated cells on day 6 between DA-5-3^GFP^, DA-5-3^GFP^-vector cells and *Thop1*-OE^GFP^ cells in BF and FITC-channel. Scale bar, 100 μm.

**Table S1. Primer Sequences**

| Experiment | Target | Sequence (5’-3’) |
| --- | --- | --- |
| qPCR | *Gapdh* | F: ATCACTGCCACTCAGAAG |
|  |  | R: AAGTCACAGGAGACAACC |
|  | *Oct4* | F: GGGATGGCATACTGTGGAC |
|  |  | R: CTTCCTCCACCCACTTCTC |
|  | *Klf2* | F: GGTAGTGGCGGGTAAGCTC |
|  |  | R: AACTGCGGCAAGACCTACAC |
|  | *Klf4* | F: GTGCCCCGACTAACCGTTG |
|  |  | R: GTCGTTGAACTCCTCGGTCT |
|  | *Nanog* | F: GCCCTGAGAAGAAAGAAGAG |
|  |  | R: CGTACTGCCCCATACTGGAA |
|  | *Stella* | F: TCCTACAACCAGAAACACTAG |
|  |  | R: GTGCAGAGACATCTGAATGG |
|  | *Irx3* | F: GCTCAATGAACACCGCAAGA |
|  |  | R: GTGATGATGGCCAACATGATCT |
|  | *T* | F: AACTGCGAGTGGGTCTGGAAG |
|  |  | R: TGGGTCTCGGGAAAGCAGTG |
|  | *Gata4* | F: GCCTGCGGCCTCTACATGAA |
|  |  | R: CAGGACCTGCTGGCGTCTTA |
|  | *Nestin* | F: AGAGAAGCGCTGGAACAGAG |
|  |  | R: AGGTGTCTGCAACCGAGAGT |
|  | *Rex1* | F: TTCTTGCCAGGTTCTGGAAGC |
|  |  | R: TTTCCCACACTCTGCACACAC |
|  | *Sox2* | F: GGCGGCAACCAGAAGAACAG |
|  |  | R: GTTGCTCCAGCCGTTCATGTG |
|  | *Lin28a* | F: GTGGACGTCTTTGTGCACCAG |
|  |  | R: GCCGTCGCTCACTCCCAATA |
|  | *Smpd4* | F: CAGGGACGTGTGAACCCTGT |
|  |  | R: GTCGGGGGAAACTGGACCTT |
|  | *Thop1* | F: GAGCGCACAGCAGATCAGAG |
|  |  | R: TTGTTCGGAGACACGTGCTG |
|  | *Igfbp4* | F: CACATTGATGCACGGGCAAG |
|  |  | R: CAGCTCGCTCTGGCAAGAA |
|  | *Fam222b* | F: GACGGCGGCTGTGATTGAGA |
|  |  | R: AGCGTTCATCTGCGTGTGAG |
|  | *Nfic* | F: GGACCTGTACCTGGCCTACTT |
|  |  | R: TGGCTGGGTTCAGGTCGTATG |
|  | *Pth1r* | F: TGTGACAAGCTGCTCAAGGAAG |
|  |  | R: GGGTAGAACTTTCCCGATGCC |
|  | *Plec1* | F: GCCTGTCTGCCATCTACTTGGA |
|  |  | R: CTTCAGGTGCTCCTCGTGTG |
|  | *Inhbb* | F: TGAGATCATCAGCTTTGCAG |
|  |  | R: CTGCACCACAAATAGGTTCT |
|  | *Lrfn3* | F: AGGCATCCGCATGTACCAGAT |
|  |  | R: CACAGATCATAGGTACGGCCTG |
|  | *Mafk* | F: GAGTGCGTGTCTACAGGAGCC |
|  |  | R: TCAATGTCTTGTTGGGCTTGGG |
| Splinkerette PCR | Adaptor  Adaptor | Top: GTTCCCATGGTACTACTCATATAATACGACTCACT ATAGGTGACAGCGAGCGCT |
|  |  | Bottom: GCGCTCGCTGTCACCTATAGTGAGTCGTATTA  TAATTTTTTTTTCAAAAAAA |
|  | Adaptor | R1: GTTCCCATGGTACTACTCATA |
|  | PB5’ | F1:GATATACAGACCGATAAAACACATGCGTCA |
|  | PB3’ | F1: GACGGATTCGCGCTATTTAGAAAGAGAG |
|  | Adaptor | R2: TAATACGACTCACTATAGG |
|  | PB5’ | F2: ACGCATGATTATCTTTAACGTACGTCAC |
|  | PB3’ | F2: CATGCGTCAATTTTACGCAGACTATC |
| Reverse PCR | Left 1 | F: CCTCGATATACAGACCGATAAAACA |
|  |  | R: CAAGGCCTACTAGTATTATGCCCAGT |
|  | Right 1 | F: GGTCATAGGGCCGGGATTC |
|  |  | R: GACTGAGATGTCCTAAATGCACAGC |
|  | Left 2 | F: CATGATTATCTTTAACGTACGTCACAAT |
|  |  | R: GTACATGACCTTATGGGACTTTCCTAC |
|  | Right 2 | F: TCTCCTCCACGTCACCGC |
|  |  | R: GAGCAATATTTCAAGAATGCATGCGTC |
| Plasmid construction | *Rex1* sgRNA | 1-1: caccgTCCCTGCCTTTGCATGAGTT |
|  |  | 1-2: aaacAACTCATGCAAAGGCAGGGAc |
|  |  | 2-1: caccgCCCAGGCTGAGTGGTCTTAC |
|  |  | 2-2: aaacGTAAGACCACTCAGCCTGGGc |
|  | Rat-*Rex1*-5’ arm | F: ATCTAAGGCAGGTGTTTGCC |
|  |  | R: ttGTCGACttACCGGTccACTAGTGCATTTCTTCCCTGCCTT |
|  | Rat-*Rex1*-3’ arm | F: aaACCGGTAAGTCGAGACAGATTGTCCCA |
|  |  | R: ttGTCGACCTGAGTGGACATGTATGTTG |
|  | *Thop1* sgRNA | 1-1: caccgCCACCGTGAATCATCTGCGC |
|  |  | 1-2: aaacGCGCAGATGATTCACGGTGGc |
|  |  | 2-1: caccgGTACGACCGTGTGGGCGCCC |
|  |  | 2-2: aaacGGGCGCCCACACGGTCGTACc |
| Genotype | *Rex1*-genotype | F1: CCGCTGCCTATAAGCCATTGA |
|  |  | R1: CCGGACACGCTGAACTTGTG |
|  |  | F2: GATCACTCTCGGCATGGACGA |
|  |  | R2: TGACCCTCTTCCCCCATTTCC |
|  | Rat-*Thop1*-G | F: CATCGTTACCTCAGCGGGTC |
|  |  | R: TTGTTTGAGCCGGCACCTGG |
